# The relationship between growth mindset and mathematics achievement in Chinese middle school students: The role of achievement goals and grit

**DOI:** 10.1186/s40359-026-05153-6

**Published:** 2026-07-09

**Authors:** Jia Dong, Xiqing Yang, Xi Yang, Dingliang Tan, Kexin Wang

**Affiliations:** 1 School of Educational Sciences, Nanjing Normal University, Nanjing, 210097 China; 2 High school affiliated to Nanjing normal university Jiangning campus, Nanjing, 211102 China; 3 Psychological Health Center, Jingling Institute of Technology, Nanjing, 211169 China; 4 School of Psychology and Cognitive Science, East China Normal University, Shanghai, 200062 China; 5 Students’ Affairs Office, Baicheng Normal University, Baicheng, 137000 China

**Keywords:** Growth mindset, Achievement goal, Grit, Mathematics achievement

## Abstract

**Background:**

Research on the relationship between growth mindset and mathematics achievement is heterogeneous and even contradictory, especially in China. Previous studies have typically directly analyzed the relationship between the two factors, rather than exploring the impact of growth mindset on mathematics achievement through mediating mechanisms. The current study aimed to explore the relationship between growth mindset and mathematics achievement, as well as the chain mediating roles of achievement goals and grit.

**Methods:**

This study used a cross-sectional design with convenience cluster sampling. All first-grade students (Grade 7) from a public middle school in China, were invited to participate, resulting in a final sample of 691 participants. They completed a questionnaire assessing growth mindset, achievement goals, and grit. Their mathematics test scores were also collected. Statistical data analysis was performed using SPSS 27.0, including an analysis of the chain mediation effect through the PROCESS program developed by Hayes.

**Results:**

A growth mindset was found to be significantly negatively correlated with mathematics achievement, yet it also demonstrated indirect associations through achievement goals and grit. Specifically, it was positively linked to achievement via mastery-approach, mastery-avoidance, and performance-approach goals, as well as directly through grit, whereas a negative indirect association emerged through performance-avoidance goals. Furthermore, a growth mindset exhibited positive sequential indirect relationships through multiple pathways: it positively influenced achievement via mastery and performance-approach goals, which in turn supported grit; notably, it also contributed positively through a pathway in which it was negatively associated with performance-avoidance goals, which themselves were negatively related to grit, with grit ultimately correlating positively with mathematics achievement.

**Conclusion:**

The relationship between a growth mindset and mathematics achievement is primarily indirect, operating through a chain of motivational and behavioral mediators. Although a direct negative correlation was observed, growth mindset demonstrated positive sequential indirect associations with achievement via achievement goals and grit. These findings support a sequential motivational model in which mindset shapes motivational processes, which in turn drive goal-directed behaviors to produce outcomes. They also suggest that interventions targeting both adaptive goal-setting and the cultivation of grit may be more effective in promoting academic development than those focusing solely on mindset.

## Introduction

According to growth mindset theory, individuals with a growth mindset believe that intelligence can be improved and tend to demonstrate better academic performance [1–3], particularly in mathematics [4, 5]—a domain often perceived as reliant on innate talent due to its abstract and logical nature [6]. However, the empirical relationship between growth mindset and academic achievement is inconsistent [7–9]. While some studies report a positive association with mathematics achievement [4, 10] and implicate a fixed mindset in poorer performance [11], others find no or even a negative correlation [9, 12–14]. This heterogeneity appears particularly pronounced in Chinese cultural contexts, where the mechanisms may differ [9, 15, 16], yet direct empirical studies are limited and inconclusive. This unresolved ambiguity highlights the need to look beyond direct associations. This pattern of inconsistent direct relationships suggests that the link, if it exists, is likely indirect and mediated by other factors [17]. Therefore, to reconcile the conflicting evidence, research must focus on the specific pathways of association between growth mindset and academic outcomes [9, 18].

Guided by the social-cognitive model of achievement motivation, this study proposes two key mediators: achievement goals and grit. Mindset shapes the types of goals individuals adopt: a growth mindset fosters mastery-oriented goals focused on competence development, whereas a fixed mindset often aligns with performance goals focused on validation [1, 5, 19]. These goal orientations, in turn, are critical determinants of engagement and performance in mathematics [20–23]. Furthermore, mindset may influence sustained effort: individuals with a growth mindset, less likely to be discouraged by failure or setbacks, are theorized to exhibit greater grit—perseverance and passion for long-term goals—which directly supports academic achievement [24, 25].

Notably, the mediators in question may function not merely in parallel but sequentially. According to the (MIE) model proposed by Burnette et al. [26], enduring changes in outcomes (e.g., academic achievement) unfold through a recursive sequence. specifically, it posits that mindsets influence motivational processes, and these processes subsequently drive goal-directed behaviors to produce outcomes. Inspired by this theoretical framework, we hypothesize that in the present context, a growth mindset first promotes adaptive achievement goals—conceptualized as a core motivational process. These goals, in turn, foster sustained effort and perseverance, captured by the construct of grit as a key behavioral propensity. Examining this sequential pathway from growth mindset, through achievement goals, to grit is therefore theoretically critical. It allows for a test of a concrete and integrated motivational sequence—tracing the progression from core belief, to strategic aim, to sustained action—that elucidates the mechanism by which mindset ultimately influences learning outcomes.

Therefore, this study aims to investigate the impact of a growth mindset on mathematics achievement among Chinese middle school students by specifically testing a hypothesized chain-mediation model in which achievement goals and grit serve as sequential mediators. This approach moves beyond examining bivariate relationships to test the specific sequential mediating pathway among these variables. By doing so, it not only tests the applicability of mindset theory in the Chinese context but also provides a precise, mechanism-based explanation for the mixed prior findings. Ultimately, clarifying this mechanism offers an evidence-based foundation for designing targeted interventions that aim to optimize students’ academic outcomes by supporting adaptive motivational and behavioral processes.

## Literature review

### The relationship between growth mindset and mathematics achievement

Growth mindset theory proposes that individuals hold different beliefs about the malleability of intelligence: those with a growth mindset view it as improvable through effort, whereas those with a fixed mindset see it as unchangeable [1]. Although the foundational theory posits that a growth mindset should foster better learning outcomes [2], the results of the empirical research reveal a complex and often inconsistent association specifically with mathematics achievement [9, 27].

In the Programme for International Student Assessment study conducted by the Organisation for Economic Co-operation and Development (OECD), students who reported having a growth mindset score better in mathematics on average than students with a fixed mindset even after accounting for student and school socioeconomic profile on average across OECD countries (difference of 18 score points) and in 57 countries and economies. However, in Hong Kong, China, the situation is quite the opposite. Students who reported having a growth mindset performed worse in mathematics compared to those with a fixed mindset, with a difference of 8 score points [28]. This is not an isolated case. There are other studies in China indicated that growth mindset is either not correlated or is negatively correlated with mathematics achievement. For example, a longitudinal study tracking 266 Chinese middle school students from Grade 7 to Grade 9 showed that students’ growth mindset did not directly predict their mathematics achievement. Another longitudinal study focusing on Chinese children aged 9 to 13 indicated that the growth mindset was unrelated to children’s initial mathematics achievement, as well as to subsequent improvements in their mathematics achievement [14, 18]. Simultaneously, other research has identified a significant positive correlation between growth mindset and mathematics achievement in Chinese samples [29], underscoring the heterogeneity of findings in this domain. Scholars have suggested that this inconsistency may be rooted in cultural context, particularly within settings such as China, where diligent effort is traditionally esteemed and students are strongly encouraged to devote themselves to academic work [30]. This cultural emphasis, however, does not imply that growth mindset is unrelated to academic outcomes, rather, it suggests that the relationship between the two may operate through more complex and nuanced mechanisms within specific cultural environments [9].

Therefore, in the Chinese cultural context, the mechanism through which a growth mindset affects mathematics achievement remains to be further elucidated through theoretical and empirical research. In conclusion, the preceding analysis leads us to propose the following hypothesis:

- H1 Growth mindset has no direct effect on mathematics achievement.

### The mediating role of achievement goals

Achievement goals refer to plans for cognitive processes, encompassing cognitive, affective, and behavioral aspects [3]. Initially, achievement goals were categorized into mastery goals and performance goals. Subsequently, on the basis of logical reasoning and empirical research, Elliot and others divided achievement goals into four dimensions, mastery-approach goals, mastery-avoidance goals, performance-approach goals, and performance-avoidance goals, according to two elements of approach and avoidance [31, 32]. Under mastery-approach goals, individuals focus on task mastery, learning, and understanding knowledge; under mastery-avoidance goals, individuals are concerned with how to avoid misunderstanding or missing knowledge; under performance-approach goals, individuals are concerned with how to surpass others to prove their abilities; under performance-avoidance goals, individuals are concerned with how to avoid appearing incompetent [32]. Theoretically, mastery-approach goals may lead individuals to view difficulties as opportunities for learning and growth, and are linked to positive adaptive outcomes. Mastery-avoidance goals may result in excessive, anxiety-driven efforts. Performance-approach goals can promote persistence in the face of challenges, though such persistence is often short-term and unstable. Performance-avoidance goals tend to evoke high anxiety and fear when encountering difficulties, making individuals more prone to maladaptive strategies such as task avoidance, effort withdrawal, or self-handicapping [32, 33]. Based on the results of empirical research, there is a relatively consistent conclusion regarding the impact of mastery-approach goals and performance-avoidance goals on mathematical learning: mastery-approach goals have a positive predictive effect on mathematical learning, including the positive prediction of math achievement [34]. In contrast, performance-avoidance goals tend to have a negative predictive effect on mathematical learning, such as leading to higher levels of math anxiety [35], lower math achievement, and more frequent use of self-protective coping strategies [21]. However, the research on the effects of mastery-avoidance goals and performance-approach goals on mathematical learning shows inconsistent results. Some studies have found a negative correlation between mastery-avoidance goals and math achievement [36], leading to higher math anxiety [35]. Other research indicates that mastery-avoidance goals positively predict a more positive learning attitude [37]. The research on performance-approach goals follows a similar pattern. Some studies have found no correlation between performance-approach goals and certain positive outcomes (e.g., intrinsic motivation [38] and challenge-seeking [39]), and even lead to negative learning attitudes [37]. However, other studies have found a positive correlation between performance-approach goals and math achievement [21, 34].

The theory of growth mindset posits that individuals form different achievement goals because they possess one of two distinct mindsets: a growth mindset or a fixed mindset. Individuals with a growth mindset believe their intelligence or abilities can be developed; consequently, they typically strive to enhance their intelligence or abilities through effort, leading to the formation of mastery goals. Conversely, individuals with a fixed mindset, believing their intelligence or abilities are at a fixed level, do not endeavor to change their intelligence or abilities, but aim to prove their capabilities, tending to focus on the formation of performance goals, with the aim of receiving positive evaluations of their abilities or avoiding negative evaluations. Hence, individuals form different achievement goals on the basis of their perceptions of their own intelligence or abilities [5, 19]. Numerous studies have shown a significant positive correlation between an individual’s growth mindset and mastery goals, and a significant negative correlation with performance goals [4, 40]. However, research from Chinese samples has shown that a growth mindset positively predicts both mastery and performance-approach goals, a finding less common in Western research [15]. On the basis of the above analysis, this study proposes the following hypothesis:

- H2 Achievement goals mediate the relationship between growth mindset and mathematics achievement.

### The mediating role of grit

It is difficult for us to imagine that the relationship between growth mindset and academic performance is not mediated by behaviors that are more closely related to academic performance [17]. The Integrated Growth Systems Framework (IGSF) specifies that a growth mindset must translate into concrete, effort-based learning practices to impact achievement [41]. In our model, grit can be understood as the perseverance in enacting precisely such adaptive practices. Grit refers to an individual’s persistent and enduring passion for long-term goals, embodying self-management and control over consistently pursued clear goals and interests. It comprises two components: consistency of interests and perseverance of effort. Consistency of interests refers to the tendency to maintain interest and goals over years despite failure or setbacks. Perseverance of effort is the tendency to work hard toward challenges and to maintain effort even in the face of difficulties and setbacks [24, 42]. grit is one of the noncognitive variables closely related to academic performance [42, 43]. It has been shown that grit’s predictive effect on academic achievement, including mathematics achievement remains significant even after controlling for factors such as study investment, self-efficacy, previous grades, gender, and socioeconomic status [44], particularly the perseverance of effort dimension, which significantly predicts academic performance [24, 45]. Several studies have shown that grit is positively correlated with or predicts mathematics achievement [25, 46, 47].

Dweck suggests that individuals with a growth mindset believe that their intelligence or abilities can be developed through adversity; thus, they do not tend to easily give up in the face of difficulties or setbacks, but rather adjust their state and seek effective problem-solving strategies, demonstrating strong persistence towards goals [5]. Similarly, individuals with grit persist in their pursuit of goals without easily giving up [24]. People with a growth mindset often exhibit more pronounced qualities of grit. Research indicates that a growth mindset significantly positively predicts the development of grit [48, 49]. Therefore, to test the pathway through which mindset beliefs translate into achievement, we propose hypothesis:

- H3 Grit mediates the relationship between growth mindset and mathematics achievement.

### The chain mediating effects of achievement goals and grit

To elucidate the mechanism linking growth mindset to achievement, it is essential to consider not just whether these mediators exist, but how they are organized. The Mindset Intervention Effectiveness (MIE) model [26] conceptualizes enduring outcome changes (e.g., in academic achievement) as following a recursive sequence across psychological levels: internalized mindsets (Level 1) shape motivational processes (Level 2), which subsequently drive goal-directed behaviors (Level 3), and ultimately lead to end results (Level 4). Guided by this framework, the present study investigates whether growth mindset (mindset) impacts mathematics achievement (outcome) through the sequential pathway of achievement goals (motivational process) and grit (goal-directed behavior).

Duckworth et al., who proposed the concept of grit, noted that grit specifies the “pursuit of superordinate goals of enduring significance,” and that individuals with grit do not give up or deviate from their goals, even without positive feedback [24]. It can be seen that grit is based on the pursuit of goals, so does the type of goal affect the degree of grit? The answer is affirmative. Achievement goal theory supports this view: individuals with different goal orientations show distinct patterns of persistence when facing difficulties. Those with mastery-approach goals view challenges as opportunities for growth and demonstrate the highest and most stable persistence. While individuals with mastery-avoidance goals exert effort, their anxiety about not fully mastering tasks leads to a more complex persistence pattern. Those with performance-approach goals exhibit strong initial persistence that often declines after failure, whereas individuals with performance-avoidance goals are most likely to disengage or resort to self-handicapping to avoid negative evaluation [31]. Empirical evidence aligns with this pattern, indicating that students with mastery goals persist the longest in the face of task failure, students with performance-approach goals initially exhibit high persistence that decreases after failure, and students with performance-avoidance goals show the most pronounced tendency to give up [50]. Furthermore, research confirms that mastery goals significantly positively predict grit, while performance-avoidance goals significantly negatively predict grit [51, 52]. Therefore, we propose H4:

- H4 Growth mindset is associated with students’ mathematics achievement through the chain mediating effects of achievement goals and grit.

## Methods

### Participants and procedure

This study employed a cross-sectional design with convenience cluster sampling to administer a questionnaire to all first-year junior high school students (Grade 7) from a school in Jiangsu Province, China, comprising 18 classes (*N* = 774). After excluding invalid questionnaires, 691 valid questionnaires were obtained, resulting in an effective rate of approximately 95.71%. Of these, 339 were male (49.06%) and 352 were female (50.94%), with an average age of 12.97 years (standard deviation = 0.49). The survey was conducted after receiving approval from the institutional ethics committee and written informed consent from the participants and their legal guardians. Data collection was carried out through paper questionnaires during regular class sessions. Before the survey, researchers explained the purpose and extent of the study to the participants, clarified that their personal information would be kept strictly confidential and that their responses would only be used for research purposes. The researchers emphasized that the choice to participate was voluntary.

### Measures

#### Growth mindset

The Implicit Theories of Intelligence Scale for Children was devised by Dweck et al. specifically for adolescents [1]. It comprises six items, including three items reflecting a growth mindset and three reflecting a fixed mindset. The three fixed mindset items can be used independently to minimize the acquiescence bias. In this study, we utilized these three fixed mindset items, scoring them on a Likert scale from 1 (strongly disagree) to 5 (strongly agree). Items were reverse scored and summed, with higher scores indicating a stronger inclination toward a growth mindset. The Cronbach’s alpha for this scale in the current study was 0.92. 

#### Achievement goals

The Achievement Goal Questionnaire-Revised was developed by Elliot and colleagues in 2001 and revised in 2008 [53]. This questionnaire assesses four dimensions: mastery-approach goals, mastery-avoidance goals, performance-approach goals, and performance-avoidance goals, with three items per dimension. Responses are recorded on a Likert scale from 1 (strongly disagree) to 5 (strongly agree), with higher scores on each dimension indicating a stronger tendency toward that particular goal orientation. The Cronbach’s alpha value for this questionnaire in the present study was 0.80. 

#### Grit

The Grit Scale was created by Duckworth in 2007 [42] and consists of two dimensions: consistency of interests and perseverance of effort, with a total of 12 items. This scale uses a Likert scale to score responses from 1 (strongly disagree) to 5 (strongly agree), in which higher scores indicate a higher level of grit. The Cronbach’s alpha value for this scale in the current study was 0.63. 

#### Mathematics achievement

Students’ mathematics achievement was measured by their scores on the Grade 7 standardized final examination administered in a city in Jiangsu Province, China (range = 0–100, *M* = 87.36, *SD* = 10.36). Developed by a local panel of mathematics assessment experts, the examination ensured high content validity and alignment with national curriculum standards. It comprehensively covered core Grade 7 mathematics domains, including rational numbers, linear equations, algebraic expressions, and introductory geometry. The test consisted of computational questions assessing procedural fluency and proof-based questions evaluating logical reasoning abilities.

### Statistical analysis

This study utilized SPSS 27.0 software for statistical data analysis. This included tests for common method bias, descriptive statistics, correlational analyses, and chain mediating effect analysis. The Bootstrap-based mediation effect test is conducted using the process program developed by Hayes, specifically applying Model 80 [54]. A bias-corrected bootstrapping test with a 95% confidence interval (CI) from 5000 resamples was adopted to calculate the indirect effects. All values in the model were standardized.

## Results

### Common method bias test

To eliminate the potential common method bias caused by questionnaire methods and single data sources, the Harman single-factor method was employed to test for common method bias in the data. The results indicated that there were five factors with eigenvalues greater than 1. The first factor explained 26.14% of the total variance, which is below the critical standard of 40%, indicating the absence of common method bias in the data in this study.

### Descriptive statistics and correlation analysis

Descriptive statistics and Pearson’s correlation analysis were conducted on growth mindset, achievement goals, grit, and mathematics achievement. The results, as shown in Table 1, revealed that growth mindset was significantly positively correlated with mastery-approach goals, mastery-avoidance goals, performance-approach goals, and grit (*r* = 0.16, 0.12, 0.15, 0.31, *p <* 0.01), and significantly negatively correlated with performance-avoidance goals (*r* = − 0.13, *p <* 0.01), but not significantly correlated with mathematics grades. Grit was significantly positively correlated with mastery-approach goals, mastery-avoidance goals, and performance-approach goals (*r* = 0.40, 0.31, 0.31, *p <* 0.01), and significantly negatively correlated with performance-avoidance goals (*r* = − 0.19, *p <* 0.01). Grit was also significantly positively correlated with mathematics grades (*r* = 0.18, *p <* 0.01). Mathematics grades were significantly positively correlated with mastery-approach goals, mastery-avoidance goals, and performance-approach goals (*r* = 0.20, 0.18, 0.18, *p <* 0.01), but not significantly correlated with performance-avoidance goals.

**Table 1:** Descriptive statistics and correlation analysis of growth thinking, achievement goals, perseverance and mathematics achievement

variable	M ± SD	1	2	3	4	5	6	7
1. gender	—	—						
2. growth mindset	10.42 ± 3.75	0.07	—					
3.mastery-approach	13.60 ± 1.66	0.08^*^	0.16^**^	—				
4. mastery-avoidance	13.22 ± 1.95	0.04	0.12^**^	0.67^**^	—			
5. performance-approach	12.97 ± 2.07	-0.03	0.15^**^	0.62^**^	0.55^**^	—		
6. performance-avoidance	11.14 ± 2.58	-0.04	-0.13^**^	0.16^**^	0.26^**^	0.32^**^	—	
7. grit	39.24 ± 6.17	0.10^*^	0.31^**^	0.40^**^	0.31^**^	0.31^**^	-0.19^**^	—
8. mathematics achievement	87.36 ± 10.36	0.07	0.01	0.20^**^	0.18^**^	0.18^**^	-0.05	0.18^**^

^*^p < 0.05, ^**^p < 0.01

### Chain mediation analysis

Following the mediation effect testing procedure proposed by statisticians, mediation effect tests can be conducted regardless of whether the direct effect of the independent variable on the dependent variable is significant [55]. Therefore, the related analysis results of this study met the statistical requirements for further mediation effect testing on achievement goals and grit. The analysis involves growth mindset as the independent variable, achievement goals and grit as the mediating variables, and mathematics achievement as the dependent variable. Gender is included as a control variable because of its varying degrees of correlation with other variables.

**Fig. 1:**
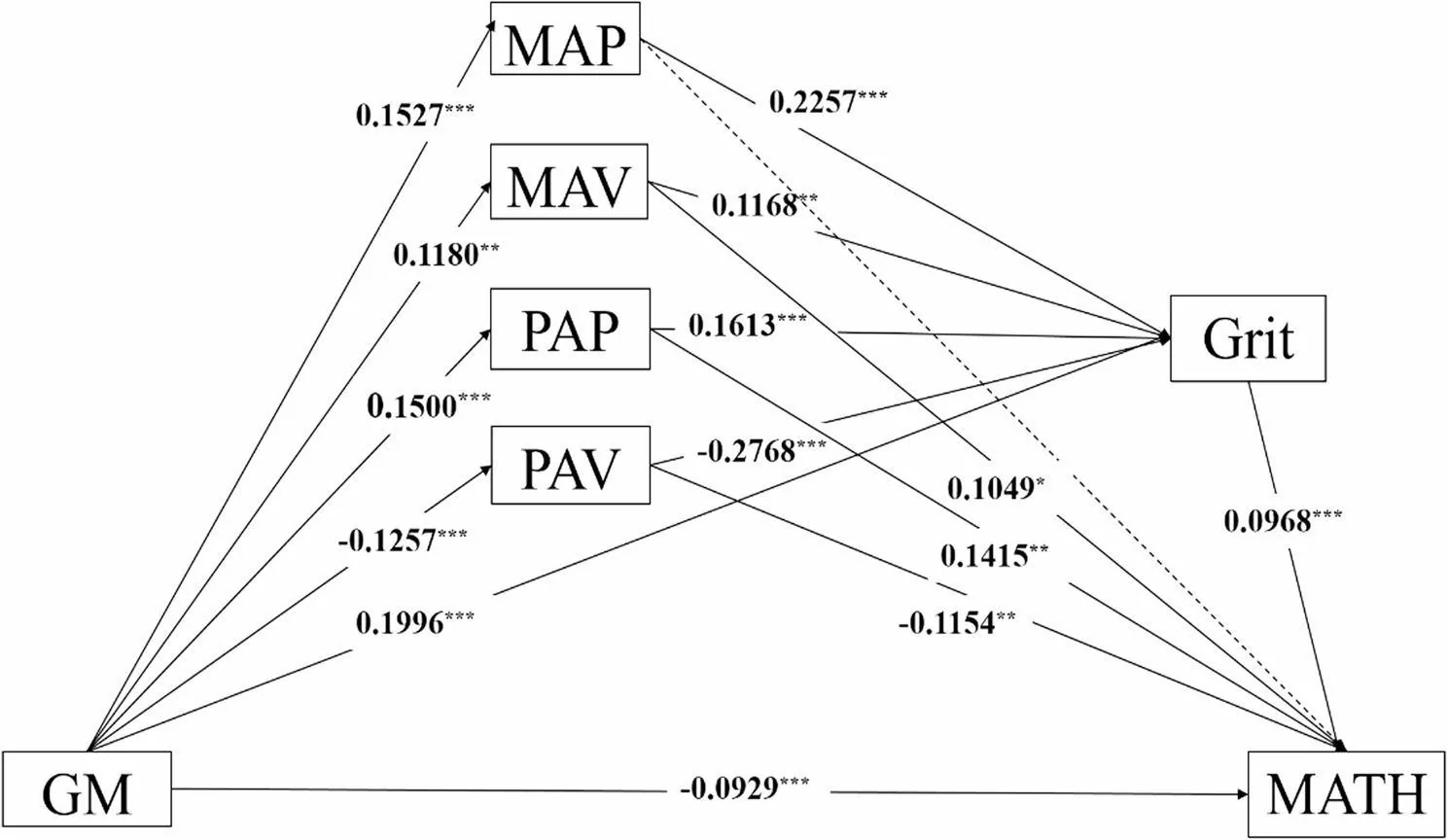
The chain mediating effect model. Note: “GM” represents growth mindset, “MAP” represents mastery-approach goals, “MAV” represents mastery-avoidance goals, “PAP” represents performance-approach goals, “PAV” represents performance-avoidance goals, and “Math” represents mathematics achievement; *p < 0.05, **p < 0.01, ***p < 0.001

As shown in Fig. 1; Table 2, which present the results of the mediation model with growth mindset as the independent variable, achievement goals and grit as the mediators, and mathematics achievement as the dependent variable, the findings are summarized as follows: growth mindset was significantly and positively associated with mastery-approach goals (*β* = 0.1527, *p* < 0.001), mastery-avoidance goals (β = 0.1180, *p* < 0.01), and performance-approach goals (*β* = 0.1500, *p* < 0.001), while it was significantly and negatively associated with performance-avoidance goals (*β* = -0.1275, *p* < 0.001). Additionally, growth mindset showed a significant positive association with grit (*β* = 0.1996, *p <* 0.001), but a significant negative association with mathematics achievement (*β* = -0.0929, *p <* 0.05). Furthermore, mastery-approach, mastery-avoidance, and performance-approach goals were all positively associated with grit (*β* = 0.2257, *p <* 0.001; *β* = 0.1168, *p <* 0.01; *β* = 0.1613, *p <* 0.001), whereas performance-avoidance goals were negatively associated with grit (*β* = -0.2768, *p <* 0.001). In terms of achievement outcomes, mastery-approach goals were not significantly associated with mathematics achievement, while mastery-avoidance goals and performance-approach goals were positively associated with it (*β* = 0.1049, *p <* 0.05; *β* = 0.1415, *p <* 0.01), and performance-avoidance goals were negatively associated with it (*β* = -0.1154, *p <* 0.01). Finally, grit was significantly and positively associated with mathematics achievement (*β* = 0.0968, *p <* 0.05).

**Table 2:** Regression analysis of achievement goals, grit, and mathematics achievement

dependent variables	Independent variables	*R* ^2^	F	β	t
mastery-approach	gender	0.0289	10.2359	0.0648	1.7194
	growth mindset			0.1527	4.0511***
mastery-avoidance	gender	0.0154	5.3918	0.0313	0.8253
	growth mindset			0.1180	3.1126**
performance-approach	gender	0.0233	8.2169	-0.0414	-1.0961
	growth mindset			0.1500	3.9702***
performance-avoidance	gender	0.0181	6.3266	-0.0346	-0.9128
	growth mindset			-0.1275	-3.3654***
grit	gender	0.2953	47.7603	0.0551	1.7001
	growth mindset			0.1996	6.0078***
	mastery-approach			0.2257	4.7275***
	mastery-avoidance			0.1168	2.5937**
	performance-approach			0.1613	3.6790***
	performance-avoidance			-0.2768	-7.9290***
mathematics achievement	gender	0.0840	8.9464	0.0695	1.8754
	growth mindset			-0.0929	-2.3882*
	mastery-approach			0.0313	0.5650
	mastery-avoidance			0.1049	2.0331*
	performance-approach			0.1415	2.8013**
	performance-avoidance			-0.1154	-2.7716**
	grit			0.0968	2.2199*

*p < 0.05, **p < 0.01, ***p < 0.001

The chain mediating effects of achievement goals and grit are presented in Table 3. The results indicated a significant total indirect effect of growth mindset on mathematics achievement (total indirect effect = 0.0829, Bootstrap SE = 0.0160, 95% Bootstrap CI [-0.80, -0.40]). In contrast, the direct effect of growth mindset was negative and significant (Standardized *β* = -0.0929, SE = 0.3088, 95% CI [-1.3438, -0.1312]). Consequently, the total effect of growth mindset on mathematics achievement was not significant (Standardized *β* = -0.0100, SE = 0.3025, 95% CI [-0.6733, 0.5146]).

**Table 3:** Chain mediation effect test and Bootstrap analysis results

Paths	Effects	SE	95%CI	
			LLCI	ULCI
Total effect	-0.0100	0.3025	-0.6733	0.5146
Total indirect effects	0.0829	0.0160	0.0521	0.1154
Direct effect	-0.0929	0.3088	-1.3438	-0.1312
GM→ MAP→ Math	0.0048	0.0100	-0.0158	0.0248
GM→ MAV→ Math	0.0124	0.0078	0.0000	0.0302
GM→ PAP→ Math	0.0212	0.0106	0.0038	0.0451
GM→ PAV→ Math	0.0147	0.0069	0.0031	0.0299
GM→ Grit→ Math	0.0193	0.0102	0.0007	0.0410
GM→ MAP→ Grit→ Math	0.0033	0.0020	0.0001	0.0080
GM→ MAV→ Grit→ Math	0.0013	0.0010	0.0000	0.0040
GM→ PAP→ Grit→ Math	0.0023	0.0015	0.0001	0.0058
GM→ PAV→ Grit→ Math	0.0034	0.0021	0.0001	0.0083

“GM” represents growth mindset, “MAP” represents mastery-approach goals, “MAV” represents mastery-avoidance goals, “PAP” represents performance-approach goals, “PAV” represents performance-avoidance goals, and “Math” represents mathematics achievement

Furthermore, eight specific indirect pathways were significant. Growth mindset was positively associated with mathematics achievement through the simple mediation of: mastery-avoidance goals (indirect effect = 0.0124, 95% Bootstrap CI [0.0000, 0.0302]), performance-approach goals (indirect effect = 0.0212, 95% Bootstrap CI [0.0038, 0.0451]), performance-avoidance goals (indirect effect = 0.0147, 95% Bootstrap CI [0.0031, 0.0299]), and grit (indirect effect = 0.0193, 95% Bootstrap CI [0.0007, 0.0410]).

Moreover, growth mindset was also indirectly associated with mathematics achievement through the serial mediation of achievement goals and grit. Specifically, significant indirect serial pathways were observed for the following chains: through mastery-approach goals and grit (indirect effect = 0.0033, 95% Bootstrap CI [0.0007, 0.0410]), through mastery-avoidance goals and grit (indirect effect = 0.0013, 95% Bootstrap CI [0.0000, 0.0040]), through performance-approach goals and grit (indirect effect = 0.0023, 95% Bootstrap CI [0.0001, 0.0058]), and through performance-avoidance goals and grit (indirect effect = 0.0034, 95% Bootstrap CI [0.0001, 0.0083]).

## Discussion

This study examined the relationship between growth mindset and mathematics achievement, along with the potential mediating roles of achievement goals and grit. The findings revealed a significant negative direct association between growth mindset and mathematics achievement. However, a positive indirect association emerged through the independent mediating roles of achievement goals and grit, as well as through their chain mechanism, resulting in a non-significant total association between growth mindset and mathematics achievement.

### The impact of growth mindset on mathematics achievement

The findings of this study revealed a significant negative direct association between growth mindset and mathematics achievement, which is inconsistent with our research hypothesis 1 but aligns with the findings from PISA 2022 [28]. In previous research, the relationship between growth mindset and academic achievement has been controversial [9]. Although theoretical and empirical studies by Dweck et al. suggest that a growth mindset can help students achieve good mathematics achievement [4, 5], but some studies have reported non-significant or negative relationship between a growth mindset and mathematics achievement, a phenomenon that exists particularly in studies with Chinese samples [14, 18, 28]. Cross-cultural research has found that when asked to write down terms related to “learning,” US individuals often mention the importance of intelligence in learning outcomes, whereas Chinese individuals rarely discuss intelligence [56]. Chinese students believe that, compared to intelligence, their academic achievement or performance in school is more malleable [57]. This cultural context may decouple the typical motivational pathway from growth mindset to achievement observed in other settings, or even create conditions where certain interpretations of a growth mindset lead to unexpected outcomes, such as the negative direct association observed here.

However, the relationship between growth mindset and mathematics achievement is multifaceted. Growth mindset represents an individual’s belief about the malleability of intelligence, a cognitive factor that influences subsequent motivational and behavioral processes. Mathematics achievement, as the ultimate outcome, is determined by a wide array of factors. The current study clarifies that the association between growth mindset and mathematics achievement operates through a more complex mechanism rather than a simple direct effect. Specifically, we identified a competitive mediation pattern: growth mindset showed a negative direct association with achievement, yet simultaneously exhibited positive indirect associations through the mediating roles of achievement goals and grit. This explains why the total association was non-significant. This finding serves as a crucial reminder that merely promoting a growth mindset may not be sufficient, and could even be counterproductive without proper guidance, for improving academic achievement. Interventions that simultaneously target the mediating variables—such as fostering adaptive achievement goals and cultivating grit—alongside growth mindset may yield more effective and positive results.

### The separate mediating role of achievement goals and grit

The results indicated that the mediating role of mastery-approach goals was not significant, while the mediating roles of mastery-avoidance, performance-approach, and performance-avoidance goals were significant. Specifically, growth mindset was positively associated with mathematics achievement through mastery-avoidance goals and performance-approach goals. In contrast, the mediating pathway involving performance-avoidance goals showed a negative association between growth mindset and performance-avoidance goals, and performance-avoidance goals themselves were significantly negatively associated with mathematics achievement. Except for the case of mastery-approach goals, these findings largely support Hypothesis 2 and are consistent with most previous studies [21, 34, 36], verifying hypothesis 2.

Chinese culture and its learning environment possess distinct characteristics. Confucian thought closely links academic achievement to an individual’s future, creating a competitive atmosphere that emphasizes social comparison. Meanwhile, the culture of filial piety encourages high educational expectations within families. In such an environment, students often strive to surpass others to meet familial expectations and reciprocate their parents’ dedication [58, 59]. Performance-approach goals represent a positive adaptation to this cultural context, as they encourage increased effort and other constructive behaviors, thereby exerting a positive effect on academic achievement [60]. In contrast, performance-avoidance goals reflect a negative reaction to the same environment. They are associated with higher test anxiety, lower academic engagement, and ultimately a detrimental impact on academic performance [61, 62].

The absence of a mediating effect for mastery-approach goals can be understood in light of the coexistence of multiple goal orientations [63]. When individuals simultaneously hold high levels of both mastery-approach and performance-approach goals, these two orientations may interact. Research shows that in contexts where achievement is linked to external rewards or evaluation, performance-approach goals may exert a stronger positive influence on performance than mastery goals [64]. This indicates that if the intensity of performance-approach goals is too high and accompanied by strong competitive pressure, the influence of mastery goals may be overshadowed or weakened, thereby diluting or making the correlation between mastery-approach goals and academic achievement statistically non-significant. This dynamic may be amplified in Chinese culture, where competition and academic pressure are widely emphasized, potentially explaining the non-significant mediating role of mastery-approach goals observed in this study.

Additionally, the current results revealed that grit mediates the relationship between a growth mindset and mathematics achievement, verifying hypothesis 3. Because individuals with a growth mindset believe that abilities can be developed through training and practice, they do not become discouraged or give up in the face of temporary setbacks and failures. Instead, they maintain a more positive belief in effort and persistence [5]. Research has shown that a growth mindset is positively correlated with grit [4, 65], Previous research has shown that students with a growth mindset show greater perseverance and sustained interest in overcoming difficulties and challenges, as well as greater academic engagement, which is beneficial for improved academic performance [66]. Therefore, students with higher levels of grit are more likely to exert sustained effort when facing academic difficulties and to commit to overcoming academic challenges, thereby achieving better academic performance.

### Chain mediating role of achievement goals and grit

The findings of this study suggest that achievement goals and grit play a sequential mediating role in the relationship between growth mindset and mathematics achievement, which is consistent with our hypothesis 4. Specifically, a growth mindset is positively related to mastery and performance-approach goals. These goals, along with grit, serve as sequential mediators in the positive association between growth mindset and mathematics achievement. Individuals with a growth mindset are more inclined to pursue self-improvement, establish mastery goals, and continue to strive to fulfill their commitment to these goals [40]. This intrinsic motivation may make individuals more resilient in the face of difficulties or setbacks, possess a higher level of grit, exhibit more academic engagement [51], and thus achieve desirable academic outcomes. This study also found a negative correlation between growth mindset and performance-avoidance goals, as well as a negative correlation between performance-avoidance goals and grit. Individuals with a fixed mindset are more likely to have performance-avoidance goals [51], showing maladaptive motivational patterns [67], are more likely to give up in the face of failure, and have lower levels of grit [68], which negatively affects academic achievement.

It is worth noting that, the current study indicates that the positive effect of a growth mindset on mathematical achievement is mediated sequentially by performance-approach goals and then by grit. In the competitive learning environment of China, individuals with a growth mindset might also seek to demonstrate their capabilities to others or affirm their excellence through comparison with others. This could be related to the Confucian philosophy and filial piety culture that values educational achievement in China [58]. And students with performance-approach goals, upon receiving positive feedback, also possess high levels of grit [50], which is likewise conducive to achieving good academic achievement. Previous research on Chinese students has also found that both mastery and performance-approach goals have a significant positive effect on grit, and grit mediates the relationship between achievement goals and students’ academic achievement [52]. This could explain why students with a growth mindset in China have performance-approach goals, possess the perseverance to pursue these goals, and ultimately achieve good academic achievement.

### Research value and limitations

This study, through constructing a chain-mediated model, explores the relationship between growth mindset and mathematics achievement within the context of Chinese culture, as well as the mediating roles of achievement goals and grit. First, it aims to clarify the underlying mechanism linking a growth mindset to academic outcomes by tracing the pathway from motivational orientations (achievement goals) to behavioral tendencies (grit). This approach helps resolve ambiguities in the existing literature regarding these associations in the Chinese educational context. Second, the findings offer practical guidance for enhancing mathematics achievement. If direct growth mindset interventions show limited effects, educators could target the identified mediators—such as fostering mastery goals and cultivating grit—to support students’ academic development.

However, the current study has several limitations that should be acknowledged. First, the cross-sectional design and sole reliance on self-report questionnaires prevent any causal inferences about the relationships among variables. Future research should employ longitudinal or experimental designs that measure constructs at multiple time points, which would allow for stronger tests of the hypothesized mediation sequence and its developmental dynamics. Second, the sample was confined to first-year middle school students from a single school, which restricts the generalizability of the findings. The cognitive, motivational, and behavioral patterns associated with growth mindset, achievement goals, and grit may vary across educational stages, school contexts, and regional cultures. Consequently, the observed relationships may not fully represent students in other grades, schools, or broader geographical and socio-cultural settings. Future studies should include more diverse and representative samples to examine the robustness of the identified mechanisms.

## Conclusions

This study concludes that the relationship between growth mindset and mathematics achievement is primarily indirect, operating through motivational and behavioral mediators. While a direct negative correlation was observed, growth mindset showed positive indirect associations via adaptive achievement goals (mastery-approach, mastery-avoidance, performance-approach) and grit. Additionally, a significant pathway was identified wherein growth mindset correlated positively with achievement by reducing performance-avoidance goals, which were in turn negatively associated with grit—itself positively linked to achievement. This specific chain of influence vividly illustrates how a core belief can sequentially alter a maladaptive motivation, thereby freeing up a key behavioral resource that ultimately supports performance. These findings demonstrate that the effect of a growth mindset unfolds in a staged process: it first shapes students’ achievement goals (the motivational level), which then directly or indirectly regulates their capacity for sustained effort (the behavioral level), culminating in academic outcomes. This underscores that mindset, motivation, and action are linked in a specific causal order, rather than operating in isolation.

Based on these findings, educational interventions should move beyond simply promoting a growth mindset and adopt a more targeted, sequential approach. First, to maximize positive outcomes, interventions should actively foster mastery-approach and performance-approach goals while specifically reducing performance-avoidance goals. This can be achieved through classroom practices that value learning progress and self-improvement over social comparison, and by reframing challenges as opportunities rather than threats of failure. Second, since grit serves as a critical behavioral bridge linking these goals to achievement, strategies to cultivate sustained effort and perseverance—such as long-term project-based learning and training in self-regulation—should be integrated directly with goal-setting activities. Ultimately, combining mindset shaping, motivational guidance, and behavioral support in a coherent program will more effectively translate students’ beliefs into sustained academic growth.

## Data Availability

The datasets generated and analyzed during the current study are available from the corresponding author.
